# Proofreading in Young and Older Adults: The Effect of Error Category and Comprehension Difficulty

**DOI:** 10.3390/ijerph121114445

**Published:** 2015-11-13

**Authors:** Meredith A. Shafto

**Affiliations:** Department of Psychology, University of Cambridge, Downing Street, Cambridge CB2 3EB, UK; E-mail: mshafto@csl.psychol.cam.ac.uk; Tel.: +44-(0)1223-766458; Fax: +44-(0)1223-766452

**Keywords:** aging, reading, proofreading, error detection, spelling, grammar, verb agreement, semantic errors, semantic illusions, antonyms

## Abstract

Proofreading text relies on stored knowledge, language processing, and attentional resources. Age differentially affects these constituent abilities: while older adults maintain word knowledge and most aspects of language comprehension, language production and attention capacity are impaired with age. Research with young adults demonstrates that proofreading is more attentionally-demanding for contextual errors which require integration across multiple words compared to noncontextual errors which occur within a single word. Proofreading is also more attentionally-demanding for text which is more difficult to comprehend compared to easier text. Older adults may therefore be impaired at aspects of proofreading which require production, contextual errors, or more difficult text. The current study tested these possibilities using a naturalistic proofreading task. Twenty-four young and 24 older adults proofread noncontextual (spelling) and contextual (grammar or meaning) errors in passages that were easier or more difficult to comprehend. Older adults were preserved at proofreading spelling errors, but were impaired relative to young adults when proofreading grammar or meaning errors, especially for difficult passages. Additionally, older adults were relatively spared at detecting errors compared to correcting spelling errors, in keeping with previous research. Age differences were not attributable to individual differences in vocabulary knowledge or self-reported spelling ability.

## 1. Introduction

The aging world population has bolstered research into how age affects common cognitive abilities. Proofreading is an important everyday skill which is a crucial component of reading and writing instruction (e.g., [1,2]) and a required ability in the modern workplace (e.g., [3,4]); understanding how age affects proofreading ability has implications for how we support workers and learners throughout the adult lifespan. 

In addition to being a practical skill, proofreading is also a complex cognitive function that relies on both core language processes and higher-level cognitive abilities related to attention and problem solving [5,6]. Normal aging differentially affects language knowledge, comprehension and production processes [7], and attention (see for review, [8,9,10]). However, it is not clear whether or how age affects the interaction of these component processes during proofreading as there is virtually no research examining proofreading in older adults (but see, [11]. The current study provides an investigation of naturalistic proofreading abilities in a group of cognitively healthy young and older adults, in order to identify aspects of proofreading which do and do not decline with age. 

### 1.1. Proofreading and the Input/Output Asymmetry

During proofreading, detecting and correcting errors relies on language comprehension and production, respectively. A well-documented pattern in language and aging research is the “input/output asymmetry,” that older adults tend to be well-preserved at tasks involving language input (*i.e*., perception and comprehension), but are impaired at tasks relying on output (*i.e*., production) [12,13,14,15,16]. During comprehension, older adults remain as good as young adults at extracting the meaning of sentences [17,18,19], building online syntactic structure [20,21] and updating situation models [22,23,24,25]. In contrast, during production, older adults have more word-finding failures and dysfluencies in speech [26,27,28], construct less syntactically-complex sentences [29,30] and produce language with lower idea density [31,32]. 

The input/output asymmetry has been demonstrated in the context of spelling error detection (input) and correction (output), with the finding that older adults are as good as or better than young adults at detecting spelling errors, but impaired at spelling production and error correction [13,33,34,35]. However, little research exists that examines age differences in the detection and correction of other error types, such as grammar or semantic (meaning) errors. Indeed, the few studies that have examined semantic anomaly detection in young and older adults provide mixed results: one recent study demonstrated that older adults detect semantic anomalies less often than young adults [36], but a related study showed no age differences [37]. 

Moreover, while automatic comprehension processes are preserved with age, older adults may be impaired when comprehension tasks are attentionally-demanding. For example, Stine-Morrow and colleagues have conducted a series of studies examining reading in young and older adults [38,39,40,41], applying the Self-Regulated Language Processing (SRLP) model, which asserts that reading comprehension depends on the allocation of cognitive resources to a range of levels of processing. Age may affect reading comprehension due to declines in both capacity and the ability to efficiently allocate resources in response to demands; in particular, while word-level processing is automatic for young and older adults, conceptual integration is more demanding, and older adults may need to allocate relatively more resources to conceptual integration in order to achieve equivalent comprehension [40,41]. Thus, while evidence from spelling predicts that older adults’ proofreading should reflect the input/output asymmetry, with preserved error detection and impaired correction, it is not clear that this pattern will apply when error detection is more attentionally-demanding. 

### 1.2. Attentional Demands of Proofreading: Error Category and Comprehension Difficulty

Research with young adults supports the notion that proofreading accuracy is affected by attentional requirements, and the current experiment will examine two factors that influence processing demands: error category and comprehension difficulty. Although not widely researched, a consistent finding from early proofreading experiments is that accuracy is affected by the amount of textual integration required to identify the errors [42]. Noncontextual errors like spelling mistakes, which occur within a word, are easier to proofread compared to contextual errors like grammatical violations or errors in meaning, which require integration across multiple words or sentences [6,43]. Hacker *et al.* [6] examined error detection and correction separately during a proofreading task and concluded that the advantage for proofreading noncontextual (spelling) errors is not due to differences in correction demands, as spelling errors were both detected and corrected more accurately than contextual (meaning) errors. Detecting noncontextual errors is easier because the processing is automatic, a conclusion supported by evidence that noncontextual proofreading is not affected by distractions such as noise in the environment [42]. By contrast, detecting contextual errors is attentionally-demanding and performance is impaired by a noisy environment [42], improved with familiarity with the text [6] and improved with increased physiological arousal [5]. The current experiment tests the hypothesis that age differences in proofreading will be smaller for noncontextual (spelling) errors where detection relies on well-preserved language comprehension processes, but age differences will emerge for the attentionally-demanding proofreading of contextual (grammar and meaning) errors. Preliminary support for this hypothesis comes from Zabrucky *et al.* [11], who demonstrated that older adults were preserved relative to young adults at detecting “surface errors” in text and violations of general knowledge, but were worse at detecting violations of “internal consistency” in the text, especially when error detection depended on integrating non-adjacent text. 

A second factor that increases the demands of proofreading is how difficult the text is to comprehend. Although more nebulous to define than error category, in young adults proofreading is impaired by a number of factors that make comprehension more demanding, including the style of text (e.g., expository *vs* narrative) [44] and whether it is familiar or unfamiliar [44,45,46]**.** The current experiment uses the same manipulation of difficulty used by Levy [44] by including both narrative passages which are less demanding to read and comprehend, and expository passages which are more difficult to comprehend. If difficult passages are more attentionally-demanding, older adults should be more impaired when proofreading difficult compared to easy text. 

### 1.3. Knowledge and Proofreading: An Age-Related Advantage?

A final consideration in predicting age effects on proofreading is the role of underlying linguistic knowledge and experience. Older adults typically have higher vocabularies than young adults [47], particularly for low frequency words [48], and evidence from spelling error detection suggests that spelling knowledge may be better for older adults, despite poorer spelling production [13,33,35]. Additionally, by virtue of their longer lives, older adults are likely to have greater and richer general knowledge [49] and decades’ more experience with reading and comprehending text. This knowledge may well impact on proofreading ability: For example, Furnham [50] demonstrated that young adults’ proofreading accuracy depended on crystallized knowledge as well as fluid abilities. Moreover, Stine-Morrow *et al.* [39] suggest that older adults may use their increased general knowledge to compensate for decreased processing capacity during text comprehension. In addition to the role of age-related expertise, there may be differences in the language experience of young and older adults due to historical changes in educational practices or day-to-day differences in the use of skills like reading and writing. The current study examines the role of these individual differences in linguistic knowledge and experience by relating proofreading accuracy to a number of measures of vocabulary, spelling, reading, and writing. 

The current study used a naturalistic paper-and-pencil proofreading task to examine how proofreading accuracy is affected by the interaction of age with attentional demands, language comprehension and production processes, and language knowledge and experience. Given previous research on proofreading in young adults and language processing in older adults, age was expected to differentially impair aspects of proofreading that tax attentional capacity or depend on production processes; age was expected not to impair aspects of proofreading that depend on language comprehension or underlying knowledge.

## 2. Experimental Methods 

### 2.1. Participants

Participants were 24 young adults aged 18–24 (M = 20, SD = 1.38), and 24 older adults aged 64–79 (M = 71, SD = 4.16). Participants were recruited from a participant panel managed by the University of Oxford Psychology Department, and were compensated for their time and travel costs. Members of the panel include healthy adult volunteers from the University and surrounding community. All subjects gave their informed consent for inclusion before they participated in the study. Table 1 summarizes a number of participant background characteristics, including education and self-rated health, which did not differ between the groups. Because of the hypothesized role of working memory in language comprehension [see e.g., 20 for discussion], participants were given a digit span task, revealing no age differences in backwards digit span (*p >* 0.10), but a larger forward digit span in the young group, t(45) = 2.17, *p <* 0.05. Participants also provided measures of their language abilities, including vocabulary [51], and responses to a language experience questionnaire used by Mackay and Abrams [35]. The questionnaire included a series of self-report measures of spelling abilities and instruction, and time spent engaging with a range of media including reading, writing, doing crosswords, and watching television. As summarized in Table 1, young adults reported spending more time writing than older adults, t(45) = 4.39, *p <* 0.001, while older adults had higher vocabulary scores, t(46) = −5.17, *p <* 0.001, reported better spelling training in school, t(45) = −2.34, *p <* 0.05, and spent more time watching television, t(46) = −6.22, *p <* 0.001, and doing crosswords t(45) = −4.15, *p <* 0.001. Key measures of vocabulary, spelling, reading and writing will be related to proofreading performance (see section 3.4).

**Table 1:** Mean (SD) ratings of background characteristics of participants.

Characteristics	Young		Older		*T*
	Mean	SD	Mean	SD	
Age in years	20.08	1.38	70.96	4.16	−56.80 **
Education in years	15.38	1.64	15.22	4.07	0.18
Self rated health (out of 10)	7.92	1.32	7.67	1.86	0.54
Nelson-Denny Vocabulary (out of 25)	16.96	2.60	21.21	3.08	−5.17 **
Digit span: forward	7.29	0.99	6.52	1.41	2.17 *
Digit span: backwards	5.38	1.10	4.91	1.20	1.38
Self-rated spelling training (out of 10)	6.29	2.14	8.00	2.84	−2.34 *
Self-rated spelling ability (out of 9)	6.29	1.63	6.71	2.03	−0.78
Television: hours per day	0.71	0.81	2.75	1.39	−6.22 **
Crossword puzzles: hours per day	0.08	0.28	1.02	1.07	−4.15 **
Reading: hours per day	3.33	1.63	2.71	1.30	1.47
Writing: hours per day	3.25	2.09	1.13	1.01	4.39 **

* *p <*0.05, ** *p <*0.001.

### 2.2. Materials 

Materials for the main experimental task consisted of four passages of text, each 350 +/− 10 words long. These passages were adapted from text used previously in proofreading studies [44,52]. 

There were three main manipulations of the experimental passages: comprehension *difficulty*, *error category*, and error *marking*. Difficulty was manipulated across passages, with two easy and two difficult passages. Difficulty level was adopted from Levy [44,52], where easy passages were narratives from intermediate Science Research Associates reading series, and difficult passages were evidence-based descriptions of topics from advanced Psychology texts.

Each passage contained 24 errors in total, eight each in the three main error categories: spelling, grammar, and meaning. Error type was manipulated within each passage, with eight errors of each type in each passage. Spelling errors included insertion errors (errors italicized, e.g., prematurity-*prematuriety*), omission errors (e.g., brought-*broght*), letter switches (e.g., believed-*beleived*), and letter substitutions (plasticity-*plastisity*). Grammar errors included errors in verb tense agreement, number agreement, punctuation, and capitalization. Meaning errors included substitutions (sun—*moon*) and word switches (e.g., “nonsmokers” and “smokers” swapped order in a sentence). See Table 2 for excerpts of passages with example errors**.**

Finally, error marking was manipulated across passages. For half of the passages, errors were not indicated (unmarked), and in half of the passages the location of errors was indicated with ***bold italic*** type (marked). Marking errors enabled examination of error correction without the conditional need for error detection, in comparison to the unmarked condition where correction depends on successful detection. Each participant read one easy and one difficult passage with marked errors and the passages with marked errors were counterbalanced across participants. 

**Table 2:** Excerpts of easy and difficult passages with examples of spelling, grammar and meaning errors marked in bold italics.

	Easy Passage	Difficult Passage
Spelling error	There is probably no other mountain where the mountaineer is exposed to ***graeter*** danger than on Kanchenjunga…	We can dependably produce and distinguish only a small number of different letters or speech ***suonds****…*
Grammar error	The huge annual precipitation of snow on Kanchenjunga is a disadvantage, for it ***plaster*** itself on the mountain…	An accidental inversion of words or letters or sounds can ***produces*** grotesque alterations of a sentence…
Meaning error	Because of this snow that is ***never*** building up, plus the tug of gravity, these icy masses move downwards…	To stretch these ***many*** elements to cover these ***few*** needs, we are forced to combine the elements into patterns…

### 2.3. Procedure

Participants were tested individually in a sound proof room. The order of testing was: (1) consent and demographic information measures; (2) digit span task; (3) proofreading task; (4) vocabulary test and language experience questionnaire.

The proofreading task was paper-and-pencil, and participants proofread all four of the experimental passages, alternating between easy and difficult passages. The first two passages had either marked or unmarked errors, and the order and the assignment of marked/unmarked conditions to passages was counterbalanced across participants. For each passage, participants were given written instructions that were then reviewed by the experimenter. In the unmarked conditions participants were instructed to indicate (e.g., underline or circle) any word or words that contained errors and correct the error on the page as they would in everyday proofreading, either in the text, between the lines, or in the margin. For the marked conditions participants were instructed that errors in the passages would be marked with bold italic font and they should correct the errors as in the unmarked conditions. In both conditions they had seven minutes to proofread each passage to provide ample reading time compared to 1.5 minutes of proofreading time per passage given to young adults in Levy [44].

## 3. Results and Discussion

The main outcome measure was proofreading accuracy, the proportion of errors (out of 48) that were detected and corrected in the passages from the unmarked conditions. This accuracy measure was used to examine how age, error category, and comprehension difficulty impact proofreading ability. A second set of analyses used proofreading failures to compare how age affects error detection *vs* correction during proofreading. Detection failures were measured as the proportion of missed errors in the unmarked conditions, and correction failures were measured as the proportion of incorrectly corrected errors in the marked conditions. Correction failures were taken from the marked conditions because in the unmarked conditions correcting an error is conditional on detecting it, whereas in the marked conditions it is not. While correction failures for unmarked conditions were not included in the analyses in section 3.3, they were similarly affected by age, error category, and comprehension difficulty as correction failures in the marked conditions. The final set of analyses considered whether age differences in proofreading can be accounted for by age differences in language knowledge and experience. 

### 3.1. Proofreading Accuracy in Unmarked Conditions: Age, Error Category, and Comprehension Difficulty

**Figure 1:**
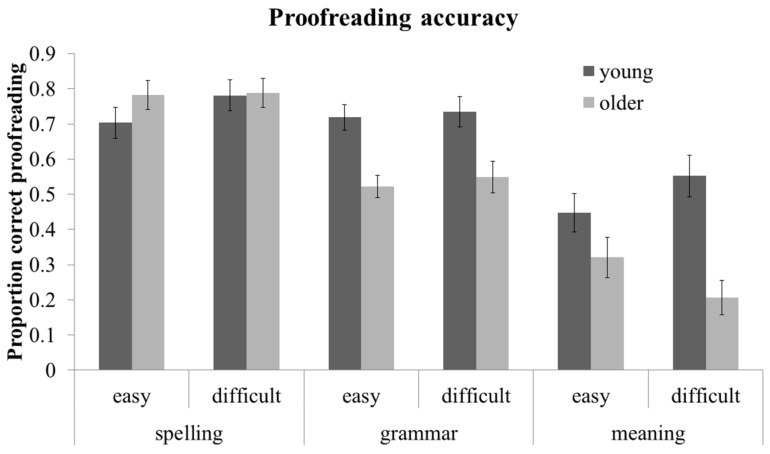
Mean proofreading accuracy **(**proportion correct proofreading for unmarked conditions) by comprehension difficulty, error category, and age. Error bars represents ±2 SEs.

Proofreading accuracy in the unmarked conditions was examined in a 2 (age: young *vs.* older) × 3 (error category: spelling, grammar, meaning) × 2 (difficulty: easy *vs.* difficult) mixed ANOVA. The results of this ANOVA are summarized in Figure 1: there was a main effect of age, F(1, 45) = 10.95, MSE = 0.110, *p <* 0.01, such that young adults were more accurate at proofreading (M = 0.66, SD = 0.13), compared to older adults (M = 0.53, SD = 0.14). There was also a main effect of error category, F(1, 45) = 95.26, MSE = 0.071, *p <* 0.001, such that proofreading was more accurate for spelling errors (M = 0.76, SD = 0.18) compared to grammar errors (M = 0.63, SD = 0.19; t(46) = 4.64, *p <* 0.001) or meaning errors (M = 0.39, SD = 0.26; t(46) = 8.66, *p <* 0.001). Additionally, proofreading was more accurate for grammar than meaning errors, t(46) = 6.38, *p <* 0.001. This pattern is in keeping with the hypothesis that proofreading contextual errors (*i.e*., grammar or meaning errors) is more difficult than proofreading noncontextual errors (*i.e*., spelling errors). The main effect of difficulty was not significant, *p >* 0.10.

These main effects were qualified by two interactions of age with error category, F(1, 45) = 13.13, MSE = 0.070, *p <* 0.01, and difficulty, F(1, 45) = 5.95, MSE = 0.026, *p <* 0.05. As can be seen in Figure 1, older adults were not impaired at proofreading spelling errors, *p >* 0.10, but were less accurate than young adults at proofreading grammar, t(45) = 4.49, *p <* 0.001, or meaning errors, t(45) =3.41, *p <* 0.01. Likewise, the effect of age was reliable for difficult passages, t(45) = 4.25, *p <* 0.001, but only marginal for easy passages, t(45) = 1.80, *p =* 0.08. Although the interaction of age and difficulty seems particularly pronounced for meaning errors (see Figure 1), the error category × difficulty and age x error category × difficulty interactions were not significant, *p*s > 0.10. 

In sum, older adults were less accurate at proofreading passages than young adults, but only for errors that require inter-word integration (grammar and meaning errors). Older adults were also differentially worse at proofreading errors in more difficult passages, but difficulty did not reliably affect proofreading meaning errors more than other error categories.

### 3.2. Comparing Marked and Unmarked Errors

The marked conditions were primarily used to isolate error correction from error detection (see section 3.3), but before examining the relevant conditions, the general effect of marking errors on proofreading accuracy was examined in a 2 (age: young *vs.* older) × 2 (marking: unmarked *vs.* marked) × 3 (error category: spelling, grammar, meaning) × 2 (difficulty: easy *vs.* difficult) mixed ANOVA. As with the unmarked condition alone (see section 3.1), there were main effects of age F(1, 45) = 22.39, MSE = 0.175, *p <* 0.001 and error category, F(1, 45) = 82.21, MSE = 0.086, *p <* 0.001, and interactions of age with error category F(1, 45) = 13.91, MSE = 0.086, *p <* 0.01 and difficulty, F(1, 45) = 7.90, MSE = 0.022, *p <* 0.01. There was a main effect of marking, F(1, 45) = 173.78, MSE = 0.045, *p <* 0.001, such that proofreading was more accurate when errors were marked (M = 0.83, SD = 0.17) than unmarked (M = 0.59, SD = 0.15). A marginal age × marking interaction, F(1, 45) = 3.93, MSE = 0.045, *p =* 0.054, indicated that marking errors may benefit young adults more than older adults, as the difference between marked and unmarked accuracy was larger for young adults (marked-unmarked difference, M = 0.27, SD = 0.12), than older adults (M = 0.20, SD = 0.13). Although the effect of marking was greater for younger adults, it was reliable for young, t(23) = 11.43, *p <* 0.001, and older adults, t(22) = 7.47, *p <* 0.001, separately. A significant marking x error category interaction, F(1, 45) = 24.96, MSE = 0.042, *p <* 0.001, indicated that there was a larger difference between marked and unmarked errors for meaning errors (marked-unmarked difference M = .37, SD = 0.26) than grammar (M = 0.19, SD = 0.16) or spelling errors (M = 0.16, SD = 0.16). However, when comparing accuracy in marked *vs* unmarked conditions, there were strong effects of marking for spelling, t(46) = 6.95, *p <* 0.001, grammar, t(46) = 8.00, *p <* 0.001 and meaning errors, t(46) = 9.74, *p <* 0.001. There were no other interactions of marking with age, error category or difficulty.

These analyses serve to verify that the manipulation of marking errors was successful at cueing error locations, improving performance across age groups, error categories, and levels of comprehension difficulty by removing the requirement to detect errors. There was some evidence that marking errors may benefit proofreading more for young adults and for meaning errors, and the next set of analyses addresses these possibilities by examining age and error category effects for error correction failures in marked conditions.

### 3.3. Detection Failures vs Correction Failures 

Error detection and correction were separated by comparing two specific proofreading failures: missed errors in the unmarked passages (detection failures), and inaccurate correction of errors in the marked passages (correction failures). 

**Figure 2:**
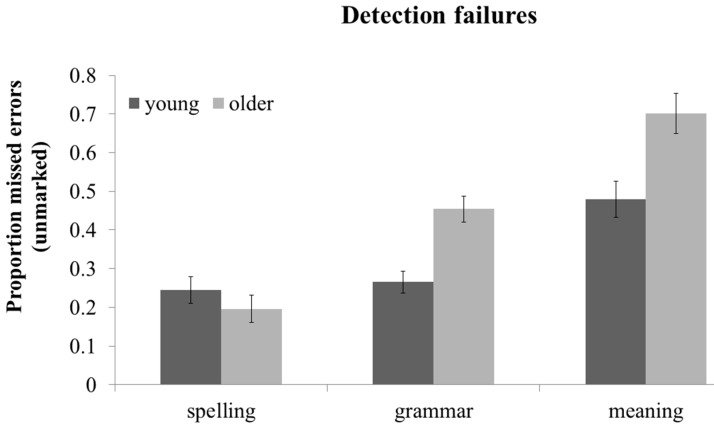
Mean detection failures (proportion missed errors in unmarked condition), by error category and age. Error bars represents ±2 SEs.

**Figure 3:**
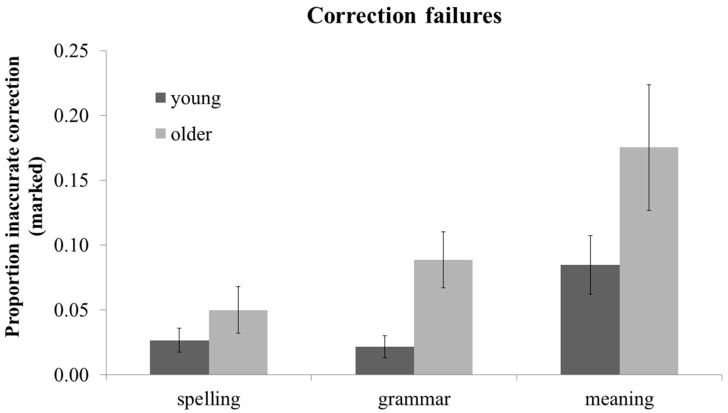
Mean correction failures (proportion inaccurate correction in marked condition), by error category and age. Error bars represents ±2 SEs.

Proofreading failures were examined in a 2 (age: young *vs.* older) × 2 (stage: detection *vs.* correction) × 3 (error category: spelling, grammar, meaning) × 2 (difficulty: easy *vs.* difficult) mixed ANOVA. Because there was no main effect of difficulty or interaction of difficulty with stage or category in this 4-way ANOVA (all ps > 0.10), in order to simplify the reporting of detection and correction failures, results are reported for the simpler 3-way 2 (age: young *vs.* older) × 2 (stage: detection *vs.* correction) × 3 (error category: spelling, grammar, meaning) mixed ANOVA. A summary of these effects can be seen in Figure 2 (detection failures) and Figure 3 (correction failures). There was a main effect of age F(1, 44) = 9.24, MSE = 0.020, *p <* 0.01, such that older adults had more failures than young adults. There was a main effect of stage, F(1, 44) = 403.05, MSE = .017, *p <* 0.001, such that there were proportionally more detection failures (M = 0.39, SD = 0.14) than correction failures (M = 0.07, SD = 0.07; See also Figure 2 and Figure 3). In keeping with measures of proofreading accuracy (see sections 3.1 and 3.2), there was also a main effect of error category, F(1, 45) = 79.78, MSE = 0.025, *p <* 0.001, with fewer failures for spelling errors than grammar errors F(1, 44) = 36.95, MSE = 0.015, *p <* 0.001, or meaning errors, F(1, 44) = 79.78, MSE = 0.058, *p <* 0.001, and fewer failures for grammar than meaning errors, F(1, 44) = 39.35, MSE = 0.053, *p <* 0.001. The age x stage interaction was marginal, F(1, 44) = 3.01, MSE = 0.017, *p =* 0.09, although age effects were reliable for both detection failures, F(1,46) = 10.03, MSE = 0.017, *p <* 0.01 and correction failures, F(1,46) = 5.37, MSE= 0.005, *p <* 0.05. There was an age x error category interaction, F(1, 44) = 9.75, MSE = 0.029, *p <* 0.01, such that the age effect was not significant for spelling errors, *p >* 0.10, but was for grammar F(1, 45) =20.26, MSE = 0.019, *p <* 0.001, and meaning errors, F(1, 44) = 8.46, MSE = .060, *p <* 0.01.

Finally, the age x stage x error category interaction reached significance, F(1, 44) = 4.14, MSE = 0.025, *p <* 0.05. This 3-way interaction was due to the interaction between age and error category being significant for detection failures, F(1, 45) = 10.80, MSE = 0.040, *p <* 0.01, but not for correction failures, *p >* 0.10. As can be seen in Figure 2, the significant age x error category interaction for detection failures was underpinned by a significant effect of age for grammar t(45) = −4.25, *p <* 0.001 and meaning detection failures t(45) = −3.17, *p <* 0.01, but no effect of age on spelling detection failures, *p >* 0.10. For correction failures, older adults performed worse than young adults across all three error types (See Figure 3), as there was an age effect for correction failures, F(1,46) = 5.37, MSE = 0.005, *p <* 0.05, but no interaction of age × error category, *p >* 0.10. 

In sum, detecting errors in the passages is clearly the most difficult and error-prone stage of proofreading, as detection failures were more common than correction failures for all error types and both age groups (see Figure 2 and Figure 3). Findings in the spelling error condition concurred with previous evidence that older adults are preserved at detecting and impaired at correcting spelling errors (e.g., [34]). Results from the grammar and meaning error conditions confirmed that older adults’ impaired proofreading in those conditions (see Section 3.1) is not solely due to problems generating corrections, as older adults were worse than young adults at both detection and correction of grammar and meaning errors. 

### 3.4. The Effect of Language Knowledge and Experience on Proofreading

The final set of analyses examined whether age effects on proofreading accuracy could be accounted for by age differences in measures of vocabulary or self-reported language experience. Four representative measures were chosen: vocabulary score, self-reported spelling ability, time spent writing, and time spent reading. These measures were related to age and proofreading accuracy in order to identify potentially relevant variables. 

First, as reported in Section 2.1 (see Table 1), older adults had higher vocabulary than young adults, young adults reported spending more time writing, and there were no age differences in self-reported spelling ability and time spent reading. Second, as summarized in Table 3, partial correlations controlling for age demonstrated that proofreading accuracy does not correlate significantly with time spent writing or reading. However, proofreading accuracy does correlate positively with both vocabulary and self-reported spelling rating. In bivariate correlations, these correlations were also significant for young adults separately, but for older adults separately only the correlation of performance and vocabulary reached significance.

**Table 3:** Pearson bivariate and partial correlations relating proofreading accuracy to vocabulary scores and self-reported spelling ability, time spent reading, and time spent writing.

	Vocabulary	Spelling	Reading	Writing
All (controlling age)	0.46 **	0.34*	0.15	0.10
Young	0.44 *	0.47*	0.18	0.11
Older	0.44 *	0.23	0.09	0.13

* *p <* 0.05, ** *p <* 0.001.

Because they were related to both age and proofreading accuracy, vocabulary and spelling ability were examined as potentially contributing to age effects in proofreading accuracy and proofreading failures. This involved repeating key analyses with vocabulary score and self-rated spelling ability as additional covariates. First, proofreading accuracy was examined in 2 (age: young *vs.* older) × 3 (error category: spelling, grammar, meaning) × 2 (difficulty: easy *vs.* difficult) mixed ANCOVAs including either vocabulary score or spelling ability as covariates. When spelling ability was included as a covariate, results were not affected. When vocabulary was included as a covariate, most findings were not affected, with the exception that the age x difficulty interaction was no longer significant, *p >* 0.10. This was because when vocabulary score was accounted for, the effect of age was significant for both easy F(1, 44) = 14.94, MSE = 0.019, *p <* 0.001 and difficult passages, F(1, 44) = 22.72, MSE = 0.020, *p <* 0.001, rather than just difficult passages (see section 3.1; see Table S1 for a summary of unadjusted and adjusted means). That is, controlling for vocabulary did not account for older adults’ poorer proofreading performance; instead, the age difference became more reliable. 

Second, detection and correction failures were examined in 2 (age: young *vs.* older) × 2 (stage: detection *vs.* correction) × 3 (error category: spelling, grammar, meaning) mixed ANCOVAs including either vocabulary score or spelling ability as covariates. As with proofreading accuracy, when spelling ability was included as a covariate, findings were not affected. When vocabulary was included as a covariate, most findings were not affected, with the exception that the age × stage and age × stage × error category interactions became nonsignificant, ps > 0.10. This was because when vocabulary score was accounted for, the effect of age in detection, F(1, 44) = 23.86, MSE = 0.014, *p <* 0.001 was similar to that in correction, F(1, 45) = 25.61, MSE = 0.003, *p <* 0.001; without including vocabulary as a covariate, age effects were reliable for both detection and correction failures, but were stronger for correction failures (see Section 3.3; see Table S2 for a summary of unadjusted and adjusted means).

In sum, while some individual differences in language experience related to proofreading ability, these effects do not account for the reported age-related declines in proofreading. Indeed, controlling for vocabulary knowledge resulted in more reliable effects of age on proofreading accuracy and failures. 

### 3.5. Discussion 

The current study contributes to the under-researched topic of proofreading in older adults, and provides a valuable step in characterising the processes underpinning proofreading that are spared and impaired in old age. At the core of the current findings is the interaction of age and error type for proofreading accuracy: older adults were just as accurate as young adults at proofreading spelling errors in text, but were less accurate at proofreading errors in grammar or meaning. These age-related impairments are in keeping with previous findings [11] and were not simply due to a lack of knowing how to correct these errors: when examining detection and correction separately, older adults were impaired at both detection and correction of grammar and meaning errors, while findings from spelling were in keeping with previous evidence of spared spelling error detection and impaired spelling error correction [13,34,35].

#### 3.5.1. Age and Proofreading: The Role of Language Knowledge and Attention

In addition to providing insight into processes underpinning proofreading, the current results also highlight the need for further research, in particular into the role of attention and language knowledge. 

The current findings support the hypothesis that older adults are more impaired at attentionally-demanding aspects of proofreading, including proofreading contextual errors (such as grammar and meaning errors) and more difficult text. In keeping with previous research [6,42,43], both young and older adults were less accurate at proofreading contextual compared to noncontextual errors, and more difficult compared to easier passages. Moreover, older adults were particularly impaired relative to young adults when proofreading contextual errors or difficult text, although difficulty and error category did not interact. However, in the current design neither age nor proofreading was directly related to independent measures of attention, which should be included in future research to directly test for the effect of individual differences in attentional capacity.

Because proofreading depends on accurate underlying knowledge [50], age-related differences in language knowledge or expertise were examined as variables which may account for age differences in proofreading. Vocabulary score reliably related to both age (older adults had higher vocabulary scores) and performance (participants with higher vocabulary scores had higher proofreading accuracy); however, controlling for vocabulary resulted in more rather than less reliable effects of age, particularly in measures of proofreading accuracy for easy passages. This finding suggests that when vocabulary scores are not controlled for, age effects may be underestimated, a possibility in keeping with evidence that older adults are preserved in using knowledge to support reading comprehension [53] and that older adults may use their greater language expertise to compensate for declines in some aspects of reading [39,54,55]. 

While older adults have greater language experience due to their longer lives, young participants may have another type of advantage in the current study: while the participant panel is open to all community members, young participants were likely University of Oxford undergraduates whose language habits reflected their status as students; for example, young adults spent more time writing than older adults (see Table 1). While writing time did not relate to proofreading performance (see Table 3), there may be other characteristics of the current task which advantage students. For example, difficult texts were drawn from Psychology textbook materials, and it is possible that some young participants were Psychology students and would be more familiar with the topics. Previous research on proofreading has also used textbook material (e.g., [11]) which may give a general advantage to student participants. Future research would benefit from a more thorough investigation of how individual differences in language knowledge and expertise influence proofreading accuracy, in particular because in the current study these measures were limited to vocabulary scores and self-report measures (of reading, writing, and spelling). These improvements, in conjunction with the use of larger and more representative samples, are important for developing ways of applying these findings to the support of older adults’ proofreading skills.

#### 3.5.2. Does Error Detection Reflect Language Comprehension?

While the current findings for detecting and correcting spelling errors are in keeping with previous evidence of the input/output asymmetry [13,33,34,35], age deficits in detecting grammar and meaning errors are more difficult to resolve with previous evidence that older adults have generally well-preserved language comprehension, including core aspects of syntactic and semantic processing see for review [7,15,16]. The current results are consistent with other evidence from reading that older adults are preserved at automatic word-level processing, but need to allocate relatively more processing resources when integrating across words [40,41]. However, older adults in those studies maintained equivalent levels of comprehension performance to young adults, and other studies suggests older adults are preserved in many aspects of online language processing, including during text comprehension [17,19,20]. It may be that proofreading introduces attentional demands that are not required for typical text comprehension. In this case, the current results provide a cautionary note about using error detection as evidence of comprehension (e.g., [11]). Especially when potential age differences are being investigated, attentional demands may introduce a confound due to age-related declines in attentional capacity; similar concerns have been raised about studies investigating age-related changes in language comprehension which introduce episodic memory demands, or working memory or attentional demands that are not typical of naturalistic language processing (see e.g., [7,15,16] for discussion). 

## 4. Conclusions

The current findings provide clear evidence that normal aging impairs some aspects of proofreading, but that the effect of age is moderated by both the type of error and the difficulty of text comprehension. These findings have implications for how we support reading and writing in later life; however, in order to develop the means of this support, additional research is needed into the roles the component representations and processes underpinning proofreading, including attention, language expertise and lifestyle factors such as current reading and writing habits. 

## Supplementary Files

Supplementary File 1
