# Supplementary material for: Proofreading in Young and Older Adults: The Effect of Error Category and Comprehension Difficulty
Source: Int J Environ Res Public Health. 2015 Nov 13;12(11):14445–60. doi: 10.3390/ijerph121114445 (PMC4661659; doi:10.3390/ijerph121114445)
Supplement: Supplementary File 1 [file ijerph-12-14445-s001.pdf]

Supplementary Information

Proofreading in Young and Older Adults: The Effect of Error Category and Comprehension Difficulty

Table S1. Means and standard errors (SE) of proofreading accuracy by age and difficulty. Means are presented unadjusted and adjusted for vocabulary score.

		Younger		Older	
		Mean	SE	Mean	SE
Easy passages	Unadjusted	0.62	0.03	0.54	0.03
	Adjusted	0.68	0.03	0.49	0.03
Difficult passages	Unadjusted	0.69	0.03	0.52	0.03
	Adjusted	0.72	0.03	0.48	0.03

Table S2. Means and standard errors (SE) of proofreading failures by age and stage. Means are presented unadjusted and adjusted for vocabulary score.

		Younger		Older	
		Mean	SE	Mean	SE
Detection failures	Unadjusted	0.33	0.03	0.45	0.03
	Adjusted	0.29	0.03	0.49	0.03
Correction failures	Unadjusted	0.04	0.02	0.11	0.02
	Adjusted	0.01	0.02	0.15	0.02

© 2015 by the authors; licensee MDPI, Basel, Switzerland. This article is an open access article distributed under the terms and conditions of the Creative Commons Attribution license (http://creativecommons.org/licenses/by/4.0/).
